# Simultaneous Determination of Salidroside and Its Aglycone Metabolite *p*-Tyrosol in Rat Plasma by Liquid Chromatography-Tandem Mass Spectrometry

**DOI:** 10.3390/molecules17044733

**Published:** 2012-04-23

**Authors:** Na Guo, Zhiwei Hu, Xiaoxu Fan, Jian Zheng, Dehui Zhang, Tao Xu, Tao Yu, Yang Wang, Haiying Li

**Affiliations:** 1 Alkali Soil Natural Environmental Science Center, Northeast Forestry University/Key Laboratory of Saline-alkali Vegetation Ecology Restoration in Oil Field, Ministry of Education, Harbin 150040, China; Email: guona0329@126.com (N.G.); huzhiwei.haerbin@yahoo.com.cn (Z.H.); 2 College of Life Sciences, Heilongjiang University, Harbin 150080, China

**Keywords:** salidroside, *p*-tyrosol, metabolite, LC-MS/MS, pharmacokinetics

## Abstract

Salidroside and its aglycone *p*-tyrosol are two major phenols in the genus *Rhodiola* and have been confirmed to possess various pharmacological properties. In our present study, *p*-tyrosol was identified as the deglycosylation metabolite of salidroside after intravenous (i.v.) administration to rats at a dose of 50 mg/kg, but was not detectable after intragastric gavage (i.g.) administration through HPLC-photodiode array detection (PDA) and liquid chromatography-tandem mass spectrometry (LC-MS/MS) analysis. Next, an accurate and precise LC-MS/MS method was developed to quantitatively determine salidroside and *p*-tyrosol in rat plasma samples. Samples were analyzed by LC-MS/MS on a reverse-phase xTerra MS C18 column which was equilibrated and eluted with an isocratic mixture of acetonitrile-water (1:9, v/v) at a flow rate of 0.3 mL/min. The analytes were monitored by multiple reaction monitoring (MRM) under the negative electrospray ionization mode. The precursor/product transitions (*m/z*) were 299.0→118.8 for salidroside, 137.0→118.9 for *p*-tyrosol and 150.1→106.9 for the internal standard (IS), paracetamol, respectively. The calibration curve was linear over the concentration ranges of 50–2,000 ng/mL for salidroside and 20–200 ng/mL for *p*-tyrosol. The inter- and intra-day accuracy and precision were within ±15%. The method has been successfully applied to the pharmacokinetic study and the oral bioavailability was calculated.

## 1. Introduction

Plant phenols often occur as glycosides, such as flavonoid glycosides, and phenylethanoid glycosides. Phenol glycosides have the potential of deglycosylation and result in its aglycone metabolites *in vivo*. The glycoside forms are hydrolyzed by β-glucosidases to the aglycone forms in the jejunum [1,2,3], and the released aglycone forms are either absorbed intact by the intestine or further metabolized by intestinal microflora into several other products before absorption [4,5].

**Figure 1:**
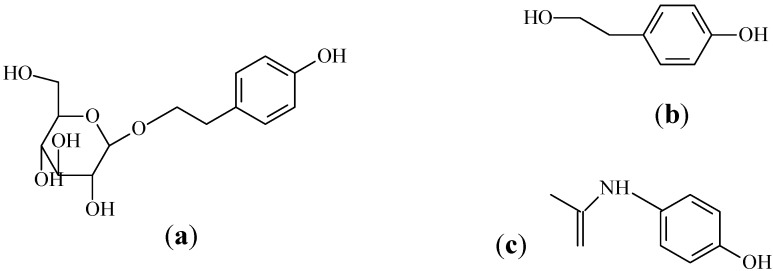
Chemical structures of (**a**) salidroside; (**b**) *p-*tyrosol; and (**c**) paracetamol (IS).

Salidroside (*p*-hydroxyphenylethyl-*O*-*β*-D-glucopyranoside, Figure 1a) and *p*-tyrosol (the aglycone of salidroside, Figure 1b) are two major phenols in the genus *Rhodiola*. Salidroside, which possesses various pharmacological properties, is used as an adaptogen in traditional Tibetan medicines, it has also shown anti-inflammation [6], resisting anoxia [7], anti-aging [8], antioxidative [9], and anti-cancer activities [10] *in vitro*, and it also has hepatoprotective [11] and cardioprotective effects [12] in rats. Furthermore, the content of salidroside is one of the criteria to evaluate the medicinal quality of *Rhodiola *[13], as well as being one of active standard components for *Rhodiola rosea* extract, which is valued as a strengthening tonic to increase physical and mental stamina and sold under different brand names on major websites (amazon.com, buy.com, and drugstore *etc.*) and in drug stores (Walgreens and GNC) in the United States. *p*-Tyrosol is also the most abundant biophenol in extra virgin olive oil [14]. It has been proven to fully protect Caco-2 cells against the cytotoxic/apoptotic effects of ox LDL [15], and to inhibit the activity of leukocyte 5-lipoxygenase in rat peritoneal mixed leukocytes [16]. Moreover, *p-*tyrosol could penetrate and accumulate in macrophages, and improve the intracellular antioxidant defense systems, even counteracting cardiovascular diseases [14].

The oral or intravenous (i.v.) administration of salidroside and *Rhodiola *extracts to rat or beagle dog and the pharmacokinetic parameters of salidroside have been reported [13,17,18,19]. The pharmacokinetic studies of *p-*tyrosol via i.v. injection have also been elucidated [20,21]. The analysis methods of salidroside have been developed and applied to plant materials and biological matrices, including liquid chromatography with ultraviolet detection (LC-UV) [18], liquid chromatography with mass spectrometry (LC-MS) [17,19], and liquid chromatography coupled with tandem mass spectrometry (LC-MS/MS) [13,22], and the simultaneous determination of salidroside and *p*-tyrosol in *Rhodiola* were established by HPLC [23,24]. However, in all these studies the metabolic characteristics of salidroside *in vivo* has not been mentioned so far.

To explore whether the deglycosylation of salidroside into *p*-tyrosol occurs *in vivo*, and to elucidate the contribution of *p-*tyrosol to the bioavailability of salidroside, in the present study intragastric gavage (i.g.) and i.v. administration of salidroside to rats were performed. The identification of salidroside and *p-*tyrosol in plasma samples was conducted by HPLC with photodiode array (PDA) detector and LC-MS/MS, respectively. The PDA detector records a full UV spectrum of the contents of the detector flow cell in real-time and provides the possibility for identification. LC-MS/MS using Electrospray Ionization (ESI) followed by two stages of mass selection: a first stage (MS1) selecting the mass of the intact analyte (parent ion) and, after fragmentation of the parent by collision with gas atoms, a second stage (MS2) selecting a specific fragment of the parent, collectively generating a multiple reaction monitoring (MRM) assay. The mass filters produce a very specific and sensitive response for the selected analyte that can be used to detect and integrate a peak in a simple chromatographic separation of the sample [25]. LC-MS/MS with MRM mode was also used for the following quantitative assay of salidroside and *p*-tyrosol. Then, a specific and rapid LC-MS/MS method was developed and validated to simultaneously determine salidroside and *p-*tyrosol with paracetamol (Figure 1c) as the internal standard (IS). The pharmacokinetic studies of salidroside in rats after i.g. and i.v. administration were carried out afterwards.

## 2. Results and Discussion

### 2.1. Identification of Salidroside and p-Tyrosol by HPLC-PDA and LC-MS/MS

**Figure 2:**
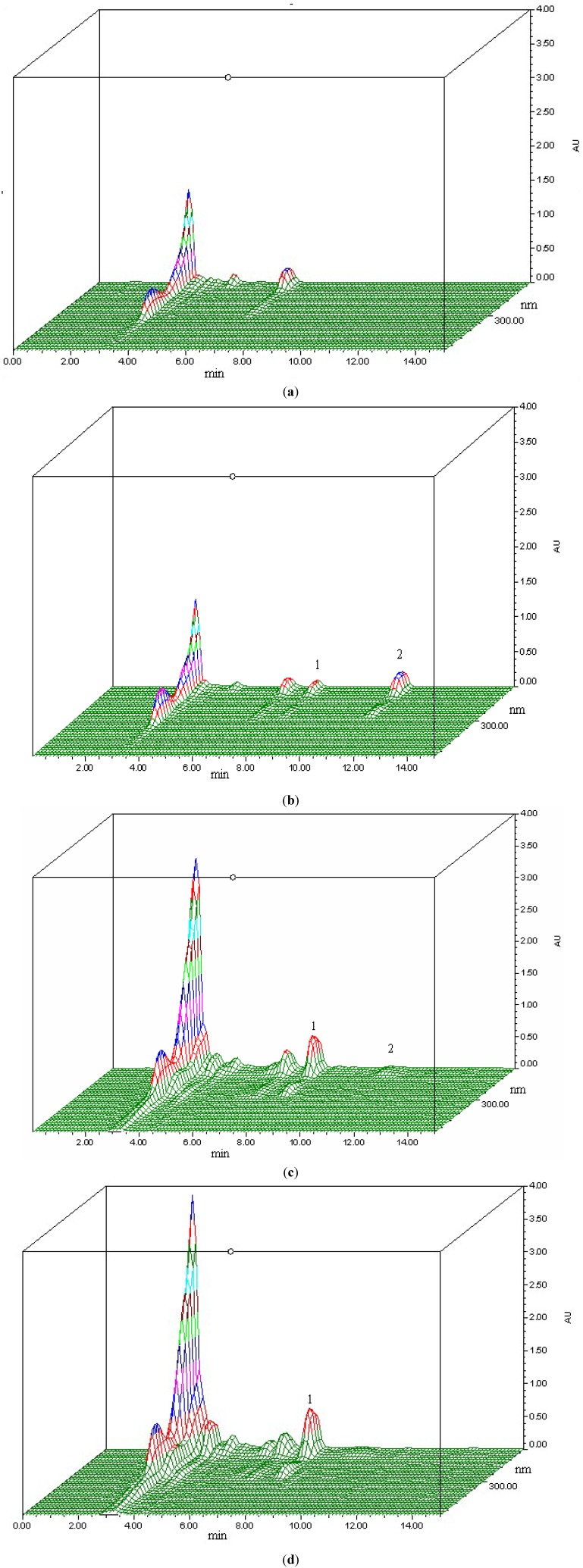
Representative chromatograms of: (**a**) a blank rat plasma sample; (**b**) a blank rat plasma spiked with salidroside and *p-*tyrosol (20 μg/mL); (**c**) a rat plasma sample collected 5 min after i.v. administration of salidroside (50 mg/kg); (**d**) a rat plasma sample collected 30 min after i.g. administration of salidroside (100 mg/kg). (1: salidroside, 2: *p*-tyrosol).

Phenol glycosides have the potential to deglycosylate and this result in its aglycone metabolite. In the present study, salidroside and the potential metabolite *p-*tyrosol, the aglycone of salidroside, were identified by HPLC-PDA and LC-MS/MS. Comparing HPLC-PDA chromatograms of rat blank plasma with those samples after i.v. and i.g. administrations, two specific peaks at the retention time of 7.68 min and 10.88 min in the samples after i.v. administration were observed, whereas only one peak at 7.68 min was shown in the samples after i.g. administration (Figure 2). UV spectra of the two peaks are similar and match to those of salidroside and *p-*tyrosol. By comparing retention times with standards of salidroside and *p-*tyrosol, peak at 7.68 min was identified as salidroside, and the peak at 10.88 min was *p-*tyrosol. 

**Figure 3:**
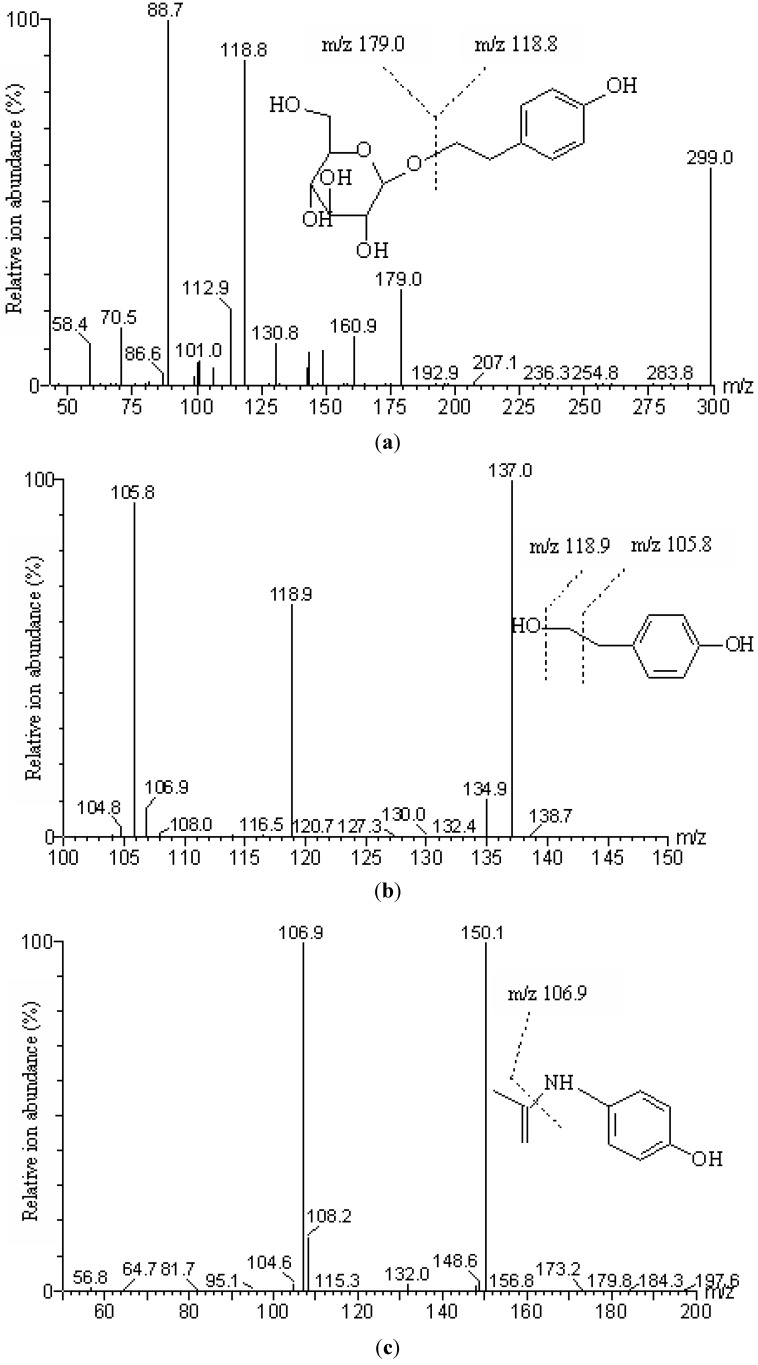
Product ion mass spectra of [M−H]^−^. (**a**) Salidroside ([M−H]^−^, *m/z* 299.0); (**b**) *p-*Tyrosol ([M−H]^−^, *m/z* 137.0); (**c**) IS (paracetamol) ([M−H]^−^, *m/z* 150.1).

The LC-MS/MS detection was performed in the negative ion mode, and the conditions for the detection of salidroside and *p-*tyrosol were optimized with the standards. The daughter ions obtained from the deprotonated molecular ions of salidroside ([M−H]-299.0) and *p-*tyrosol ([M−H]-137.0) were shown in Figure 3. The deprotonated molecular ion of salidroside produced three ions at *m/z* 88.7, 118.8 and 179.0 with higher abundance. The presumable structure of the ion at *m/z* 88.7 was [CHOHC(OH)CH_2_OH-H]^−^. The other two daughter ions at *m/z* 118.8 and 179.0 were [*p-*tyrosol-OH]^−^ and glucosyl ions, which are typical and characteristic fragments for the salidroside structure. The deprotonated *p-*tyrosol produced daughter ions at *m/z* 105.8 (loss of a -CH_2_OH fragment) and 118.9 (loss of a -OH fragment). The identification was developed by monitoring the transitions *m/z* 299.0→118.8 and 299.0→179.0 for salidroside and *m/z* 137.0→105.8, 137.0→118.9 for *p-*tyrosol, and the corresponding cone voltage and collision energy were shown in Table 1. The peak area ratio of the two coupled transitions for each analyte should be matched to the standard and also an index for the identification generated, which was 3.17 ± 0.13 for salidroside (the peak area ratio of transitions *m/z* 299.0→118.8 to *m/z* 299.0→179.0), and 3.56 ± 0.19 for *p*-tyrosol (the peak area ratio of transitions *m/z* 137.0→105.8 to *m/z* 137.0→118.9). The total run time was 3.5 min. The retention times of salidroside, *p*-tyrosol and IS were 1.94 min, 2.73 min and 1.80 min respectively. *p*-Tyrosol was detected from the samples after i.v. administration, but not from those samples obtained after i.g. administration, which further confirmed that salidroside could be metabolized to *p*-tyrosol after i.v. administration, whereas this metabolism was not detectable after i.g. administration.

The deprotonated molecular ion of paracetamol (IS) ([M−H]^−^ 150.1) only gave one characteristic product ion at *m/z* 106.9. The transitions of *m/z* 299.0→118.8 for salidroside, *m/z* 137.0→118.9 for *p*-tyrosol, *m/z* 150.1→106.9 for IS were also used for the quantification of the analytes.

**Table 1:** ESI-MS/MS parameters for salidroside, *p-*tyrosol and paracetamol (IS).

Analyte	Precursor ion ( *m/z*)	Daughter ion ( *m/z*)	Dwell time (s)	Cone voltage (V)	Collision energy (eV)
Salidroside	299.0	118.8 ^a^, 179.0	0.2	28	14
*p-*Tyrosol	137.0	105.8,118.9 ^a^	0.2	35	16
Paracetamol (IS)	150.1	106.9 ^a^	0.2	35	20

^a^ Ion for quantification.

### 2.2. Method Validation

#### 2.2.1. Selectivity and Specificity

**Figure 4:**
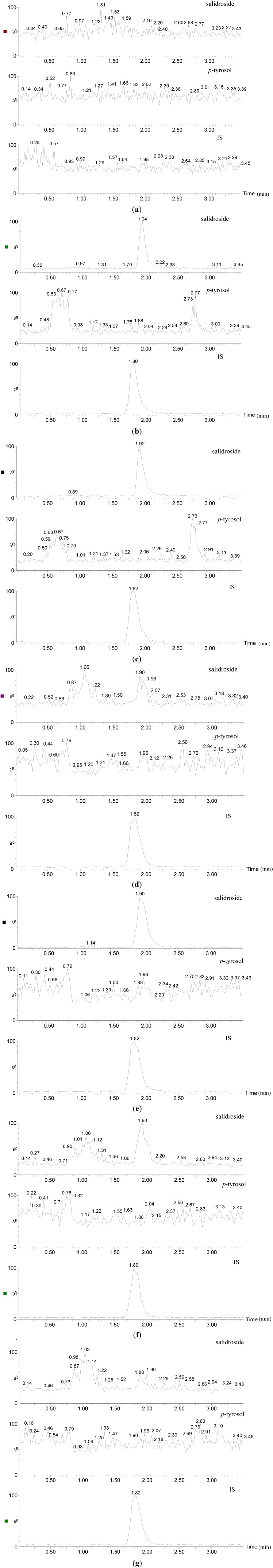
MRM chromatograms of salidroside, *p*-tyrosol and IS in (**a**) a blank rat plasma sample; (**b**) a blank rat plasma spiked with salidroside (500 ng·mL^−1^), *p*-tyrosol (100 ng·mL^−1^) and IS (200 ng·mL^−1^); (**c**) a rat plasma sample collected 5 min after i.v. administration of salidroside (50 mg/kg) with IS (200 ng·mL^−1^); (**d**) a rat plasma sample collected 5 min after i.g. administration of salidroside (100 mg/kg) with IS (200 ng·mL^−1^); (**e**) a rat plasma sample collected 30 min after i.g. administration of salidroside (100 mg/kg) with IS (200 ng·mL^−1^); (**f**) a rat plasma sample collected 2 h after i.g. administration of salidroside (100 mg/kg) with IS (200 ng·mL^−1^); (**g**) a rat plasma sample collected 4 h after i.g. administration of salidroside (100 mg/kg) with IS (200 ng·mL^−1^).

The selectivity and specificity was assessed by comparing the chromatograms of six different batches of blank rat plasma samples with the corresponding spiked plasma samples. No endogenous substances interfered with salidroside, *p-*tyrosol and the IS in any samples (Figure 4). The specificity was verified by comparing retention time of salidroside (1.94 min), *p*-tyrosol (2.73 min) and IS (1.80 min) in QC samples (n = 6). The differences were less than 1%.

#### 2.2.2. Calibration Curve, Linearity, LLOQ and LOD

The standard curves, established by plotting the ratios of the peak area of salidroside and *p-*tyrosol to that of IS with correlation coefficients >0.998, exhibited good linearity over the concentration ranges of 50−2,000 ng/mL for salidroside and of 20−200 ng/mL for *p-*tyrosol. The typical calibration equations were *y*_1_ = 0.0010 *x*_1_ + 0.0273 (*r* = 0.9987) for salidroside, and *y*_2_ = 0.0017 *x*_2_ − 0.0761 (*r* = 0.9985) for *p-*tyrosol, where *y* represents the peak area ratios of analytes to that of IS, and *x* represents the concentration of analytes. 

The LLOQ for both salidroside and *p-*tyrosol was defined as 20 ng/mL based on S/N = 10. The LOD was estimated below 10 ng/mL based on S/N = 3. Based on our preliminary experiments, 50 ng/mL for salidroside and 20 ng/mL for *p-*tyrosol could meet the needs in the pharmacokinetic studies and were selected as the lower concentrations on the calibration curves.

#### 2.2.3. Accuracy and Precision

The intra- and inter-day precision and accuracy for salidroside and *p-*tyrosol were evaluated by assaying the QC samples on six consecutive days. The results are presented in Table 2. For each QC level of salidroside, the intra-day precision was lower than 8.30%, and the accuracy was between 99.67 and 100.14%; the inter-day precision was lower than 9.99%, and the accuracy was between 99.77 and 104.17%. For each QC level of *p-*tyrosol, the intra-day precision was 10.20% or less, and the accuracy was between 104.02 and 114.82%; the inter-day precision was 11.44% or less, and the accuracy was between 103.02 and 114.29%. These data suggested that the method was accurate and precise for simultaneous analysis of salidroside and *p*-tyrosol in rat plasma samples.

**Table 2:** Accuracy and precision for the determination of salidroside and *p-*tyrosol in plasma samples (n = 6).

Analyte	Concentration (ng/mL)	Intra-day			Inter-day		
		Mean ± SD (ng/mL)	Precision (%)	Accuracy (%)	Mean ± SD (ng/mL)	Precision (%)	Accuracy (%)
salidroside	50	49.84 ± 4.14	8.30	99.67	52.08 ± 1.82	3.49	104.17
	500	500.68 ± 35.75	7.14	100.14	498.83 ± 49.83	9.99	99.77
	2,000	1,996.92 ± 62.58	3.13	99.85	2,011.80 ± 63.96	3.18	100.59
*p-*tyrosol	20	23.16 ± 2.36	10.20	114.82	22.86 ± 2.62	11.44	114.29
	100	107.08 ± 9.28	8.67	107.08	109.84 ± 2.83	2.57	109.84
	200	208.03 ± 15.80	7.59	104.02	206.04 ± 14.98	7.27	103.02

#### 2.2.4. Recovery and Matrix Effects

The recoveries of salidroside and *p-*tyrosol spiked into rat plasma were determined at three QC levels. The recoveries of salidroside were 83.98 ± 0.22%, 104.11 ± 13.29% and 99.30 ± 2.33% (n = 3) at concentrations of 50, 500, 2,000 ng/mL and those of *p-*tyrosol were 103.71 ± 3.17%, 94.67 ± 11.94% and 98.79 ± 8.70% (n = 3) at concentrations of 20, 100, 200 ng/mL, respectively (Table 3). 

The negative ion ionization was used for the detection of salidroside and *p-*tyrosol, due to the higher sensitivity than seen in the positive ion mode. The matrix effects were investigated by a post-extraction spike method in the present study. The peak area of standard analyte into spiked blank plasma was compared with the corresponding peak area obtained by directly injecting the standard analyte in the mobile phase at concentrations of 50, 500 and 2,000 ng/mL for salidroside and 20, 100 and 200 ng/mL for *p*-tyrosol in triplicate. The matrix effects were ranged from 83.91 to 113.04% for salidroside and from 80.39 to 96.07% for *p-*tyrosol, and the RSD was less than 15.0%. The results were within the acceptable limit, which indicated that ion suppression or enhancement from plasma matrices could be negligible in this study (Table 3).

**Table 3:** Recovery and matrix effects of salidroside and *p-*tyrosol in plasma samples (n = 3).

Analyte	Concentration(ng/mL)	Recovery		Matrix effects	
		Mean ± SD	RSD (%)	Mean ± SD	RSD (%)
salidroside	50	83.98 ± 0.22	0.26	113.04 ± 6.50	5.75
	500	104.11 ± 13.29	12.76	83.91 ± 5.91	7.05
	2,000	99.30 ± 2.33	3.82	89.33 ± 1.76	1.97
*p-*tyrosol	20	103.71 ± 3.17	3.05	96.07 ± 0.91	0.95
	100	94.67 ± 11.94	12.61	80.39 ± 10.54	13.11
	200	98.79 ± 8.70	8.81	90.84 ± 11.39	12.53

#### 2.2.5. Stability

The stability of salidroside and *p-*tyrosol was evaluated as described in the Experimental section. Results. shown in Table 4 and Table 5, indicated no significant degradation occurred under all storage conditions. 

**Table 4:** Stability of salidroside (n = 6).

Experimental condition	Added, C (ng·mL^−1^)	Found, C ± S.D. (ng·mL^−1^)	RSD (%)	Accuracy (%)
Standard solution2 h at RT	50	52.04 ± 1.89	3.63	104.08
	500	517.76 ± 32.17	6.21	103.55
	2,000	2,041.93 ± 12.53	0.61	102.10
Standard solution30 days at 4 °C	50	49.81 ± 4.78	0.59	99.62
	500	498.05 ± 40.72	8.18	99.61
	2,000	1,989.01 ± 69.32	3.48	99.45
QC samplesAutosampler24 h at RT	50	48.83 ± 5.37	11.00	97.67
	500	519.87 ± 29.48	5.67	103.97
	2,000	1,997.68 ± 67.16	3.36	99.88
QC samples30 days storageat −20 °C	50	51.55±1.83	3.56	103.09
	500	501.92 ± 41.15	8.20	100.38
	2,000	2,015.99 ± 52.89	2.62	100.80
QC samples3 freeze-thaw cycles	50	49.78 ± 4.78	9.60	99.56
	500	512.25 ± 28.49	5.56	102.45
	2,000	2,011.61 ± 61.51	3.06	100.58

**Table 5:** Stability of *p-*tyrosol (n = 6).

Experimental condition	Added, C (ng·mL^−1^)	Found, C ± S.D. (ng·mL^−1^)	RSD (%)	Accuracy (%)
Standard solution2 h at RT	20	21.53 ± 1.09	5.06	107.66
	100	110.95 ± 4.06	3.66	110.95
	200	218.65 ± 8.27	3.78	109.32
Standard solution30 days at 4 °C	20	23.55 ± 2.54	10.79	117.74
	100	105. 20 ± 9.55	9.08	105.20
	200	203.25 ± 13.44	6.61	101.63
QC samplesAutosampler24 h at RT	20	22.55 ± 2.73	12.09	112.73
	100	105.86 ± 12.69	11.99	105.86
	200	213.41 ± 16.64	7.80	106.71
QC samples30 days storageat −20 °C	20	22.55±2.22	9.86	112.76
	100	111.03 ± 3.32	2.99	111.03
	200	211.31 ± 16.15	7.64	105.66
QC samples3 freeze-thaw cycles	20	22.55 ± 2.23	9.87	112.76
	100	106.04 ± 10.37	9.78	106.36
	200	212.71± 13.66	6.42	106.36

### 2.3. Application to Pharmacokinetic Studies

**Figure 5:**
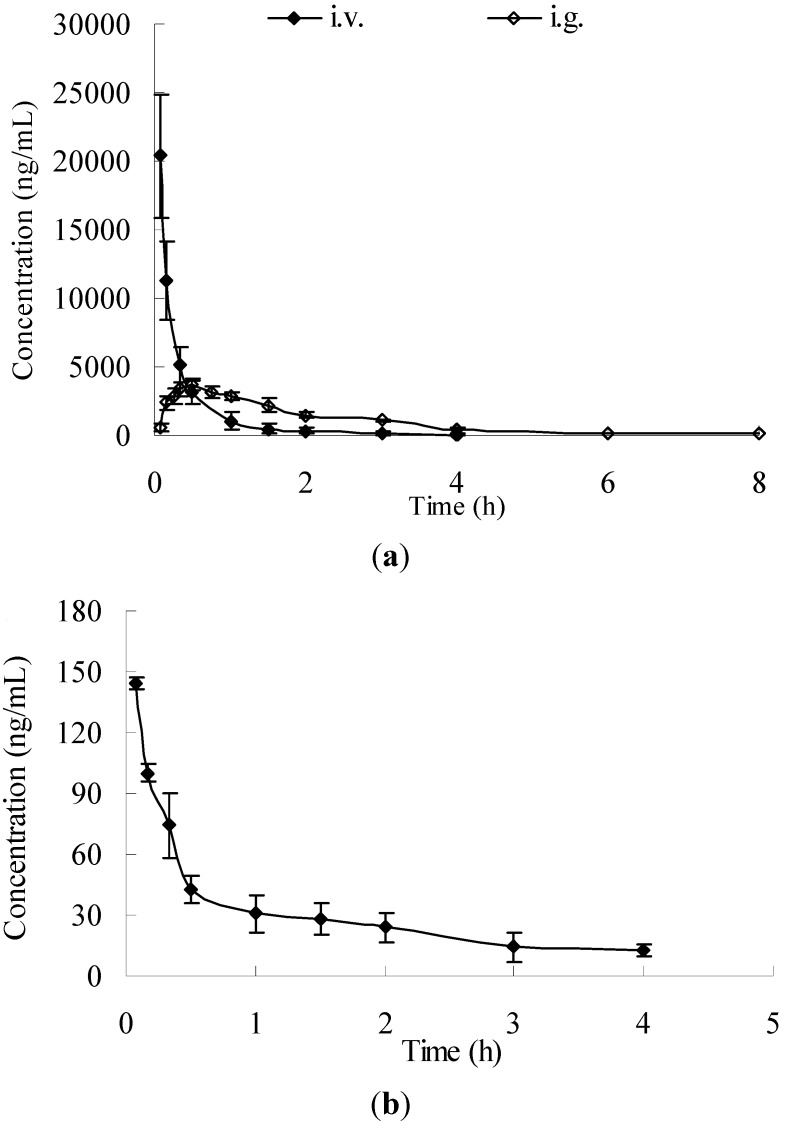
Mean concentration-time profiles in rat plasma (n = 6) obtained after i.v. administration of salidroside (i.v. 50 mg/kg) and i.g. administration of salidroside (i.g. 100 mg/kg). (**a**: salidroside; **b**: *p-*tyrosol).

The method was successfully applied to the quantification of salidroside and *p-*tyrosol in rat plasma samples after i.v. and i.g. administration. The mean concentration-time curves of salidroside and *p-*tyrosol after 50 mg/kg i.v. administration are shown in Figure 5 and the main pharmacokinetic parameters were calculated and are shown in Table 6. *p*-Tyrosol concentration reached the maximum within the first 5 min post-administration of the dose, which might be even higher than the concentration we detected, hence the T_max_ and C_max_ for *p*-tyrosol was not calculated. The t_1/2_ of elimination phase was prolonged 1.34 fold to 1.64 ± 0.30 h for *p-*tyrosol, comparing with that of 0.70 ± 0.21 h for salidroside. The AUC_0–4_ and AUC_0–∞_ values obtained were 7,060.72 ± 1,337.51 h·ng/mL and 7,135.79 ± 1,346.40 h·ng/mL for salidroside, and 122.77 ± 25.90 h·ng/mL and 146.83 ± 32.49 h·ng/mL for *p-*tyrosol, respectively, which implied that only about 2% salidroside was present as the aglycone metabolite, *p-*tyrosol, in plasma.

The mean plasma concentration-time curve of salidroside following i.g. administration was presented in Figure 5, and the pharmacokinetic parameters of salidroside were also summarized in Table 6. *p-*Tyrosol was undetectable post i.g. administration. After i.g. administration, the concentration of salidroside increased within the first 20–30 min then decreased, which meant that the absorption of salidroside reached the maximum within 20–30 min, and then the elimination was dominant. The AUC_0–8_ and AUC_0–∞_ values obtained were 7,522.82 ± 549.02 h·ng/mL and 7,724.52 ± 446.62 h·ng/mL for salidroside.

**Table 6:** Pharmacokinetic parameters of salidroside and *p-*tyrosol in rats (n = 6).

Parameters	I.v. administration (50 mg/kg)		I.g. administration (100 mg/kg)
	Salidroside (mean ± SD)	*p-*Tyrosol(mean ± SD)	Salidroside (mean ± SD)
C_max_ (ng/mL)	-	-	3,716.73 ± 860.13
T_max_ (h)	-	-	0.30 ± 0.10
T_1/2_ (h)	0.70 ± 0.21	1.64 ± 0.30	1.32 ± 0.22
AUC_0–4h_ (h·ng/mL)	7,060.72 ± 1337.51	122.77 ± 25.90	7,552.92 ± 549.02
AUC_0–∞_ (h·ng/mL)	7,135.79 ± 1346.40	146.83 ± 32.49	7,724.52 ± 446.62
MRT (h)	0.41 ± 0.13	1.84 ± 0.32	2.07 ± 0.51
Cl (L/h)	1.78 ± 0.36	54.97 ± 18.56	2.54 ± 0.15
Vss (L)	0.98 ± 0.33	0.160 ± 0.05	4.46 ± 1.19
F (%)			51.97 ± 2.67

According to the data shown in Table 6, T_1/2_ of salidroside was observed at 0.70 ± 0.21 h after i.v. administration and 1.32 ± 0.22 h after i.g. administration, which was similar to the reported T_1/2_ at 0.5 ± 0.2 h after i.v. administration and 1.1 ± 0.7 h after i.g. administration [13]. Although salidroside underwent an absorption phase after i.g. administration, which resulted in a little longer T_1/2_ (~0.6 h in difference) than that after i.v. administration, salidroside was eliminated quickly after both i.v. and i.g. administrations *in vivo*. 

The oral bioavailability in the present study was 51.97%, calculated following the equation given in the literature [26]. It was between previous results of 98% calculated at dosages of 25 mg/kg oral (p.o.) and 5 mg/kg i.v. administration [13] and 32.1% done at dosages of 12 mg/kg i.g. and i.v. administration [19] in S.D. rats, which imply that the intake of salidroside may vary upon different dosages and administrative patterns. 

Phenol glycosides are abundant in foods and medicinal herbs; as a result most research on their metabolism was under oral administration conditions. It has proven that during transfer across the jejunum and ileum, phenol glycosides are subject to extensive metabolism by phase I hydrolyzing and oxidizing enzymes, including cytochrome P450 and glucosidase enzymes and by phase II conjugating and detoxifying enzymes. The majority of aglycones, which escape metabolism in the small intestine, are subject to metabolism by phase I and II enzymes including uridine diphosphate (UDP)-glucuronosyl transferases, which lead to the formation of *O*-glucuronides, sulpho-transferases, which generate *O*-sulfates and catechol-*O*-methyl transferases, which produce *O*-methylated metabolites [27,28,29,30,31], and the aglycones may be negligibly present in the circulation [32]. In the present study, the detection of *p*-tyrosol after i.v. administration and the oral bioavailability (52.1%) reflect that salidroside may metabolize to *p*-tyrosol after i.g. administration, whereas it might be further metabolized to its sulfate or glucuronide conjugates, or even the methylate, and resulted in undetectable *p*-tyrosol in the plasma sample. Thus, more attention will be paid to the further metabolism and metabolites of salidroside and its aglycone in our further studies.

## 3. Experimental

### 3.1. Chemicals and Reagents

Salidroside was purchased from the National Institutes for Food and Drug Control (Beijing, China). Paracetamol, the IS (purity ≥ 98%) was purchased from Sinopharm Chemical Reagent Co. Ltd. (Shanghai, China). *p*-Tyrosol and HPLC-grade acetonitrile was obtained from Sigma (St. Louis, MO, USA). Ultra pure water was produced by a Millipore Milli-Q system (Billerica, MA, USA). All other reagents or solvents used were commercially available and of reagent grade. Blank rat plasma was collected from healthy Male Wistar rats weighting 200 ± 20 g (Laboratory Animal Center of Jilin University, Changchun, Jilin Province, China).

### 3.2. Chromatographic and Mass Spectrometric Conditions

The identification of salidroside and *p-*tyrosol in plasma samples was conducted by HPLC with a PDA detector and LC-MS/MS. The quantitative analysis of salidroside and *p-*tyrosol was performed on a LC-MS/MS.

The HPLC system consisted of a 1525 HPLC Pump, a 717 plus autosampler, and a 2996 PDA detector (Waters Co., Milford, MA, USA). The column used for separation was a Thermo Hypersil Gold C18 column (4.6 mm × 250 mm, 5 μm, USA). The mobile phase was a mixture of acetonitrile and water (1/9, v/v). 

The LC-MS/MS system was comprised of an Alliance 2695 HPLC and a Quattro-Micro^TM^ mass spectrometer (Waters Co.). An xTerra C18 mass column (3.5 µm, 50 × 3.0 mm, Waters Co., Milford, MA, USA) was used for separation, which was equilibrated and eluted with an isocratic mixture of acetonitrile-water (1:9, v/v) at a flow rate of 0.3 mL/min. The injection volume was 20 µL. The ESI-MS/MS detection was performed under negative ion mode under the following conditions: Capillary voltage 3.00 kV, extractor 2.00 V, RF Lens 0.1 V, desolvation gas 500 L/Hr, cone gas 50 L/Hr, source temperature 120 °C, desolvation temperature 350 °C, entrance voltage −2 V, exit voltage 1 V. ESI-MS/MS parameters were shown in Table 1. The total run time was 3.5 min. The retention time of salidroside, *p*-tyrosol and IS were 1.94 min, 2.73 min and 1.80 min respectively. Waters MassLynx 4.0 software was used for system control and data acquisition.

### 3.3. Preparation of Stock Solutions, Calibration Standard (CS) and Quality Control (QC) Samples

Stock solutions of salidroside, *p-*tyrosol and IS (paracetamol) were prepared at 200 μg/mL in acetonitrile-water (1:9, v/v), respectively, and further diluted with acetonitrile-water (1:9, v/v) to give a series of working solutions. All solutions were stored at −20 °C until use.

Calibration curves were prepared by spiking 20 μL of appropriate working solution with 100 μL of blank rat plasma. The effective concentrations were 50, 100, 200, 500, 1,000, 1,500 and 2,000 ng/mL for salidroside, and 20, 40, 80, 100, 140, 180, 200 ng/mL for *p-*tyrosol, respectively. QC samples were prepared in pool as a single batch for each concentration at concentrations of 50, 500 and 2,000 ng/mL for salidroside and of 20, 100, 200 ng/mL for *p-*tyrosol, and then divided into aliquots and stored in the freezer at −20 °C until use. The IS working solution of 250 ng/mL was diluted from stock solution as needed. The spiked rat plasma (CSs and QCs) were treated following the sample processing procedure as for the unknown samples.

### 3.4. Sample Processing

To identify salidroside and the potential metabolite *p*-tyrosol in plasma sample, an aliquot (500 µL) of plasma sample and methanol (1,500 µL) were mixed by vortex-mixing for 1 min followed by ultrasonic incubation for 10 min. After centrifuging at 45,000 g for 5 min, the clear supernatant was transferred into a new polypropylene tube and evaporated to dryness under a nitrogen stream. The residue was reconstituted in 200 µL of mobile phase. After filtering through a membrane (0.22 µm pore size), each 20 µL aliquot was injected into the HPLC-PDA or LC-MS/MS system.

The above sample preparation method was employed with minor modifications for pharmacokinetic studies. One hundred micro liters of plasma sample (blank plasma, spiked plasma or pharmacokinetics study plasma sample) and IS working solution (20 µL) was pipetted out into a 1.5 mL polypropylene tube, then methanol (280 µL) was added followed by vortex mixing for 1 min and 10 min of ultrasonic incubation. After centrifuging at 45,000 g for 5 min, the clear supernatant was transferred to a new tube and evaporated to dryness under a nitrogen stream. The residue was reconstituted in 200 µL of mobile phase. After centrifuging at 45,000 g for 10 min, an aliquot of 20 µL was injected into the LC-MS/MS system. When the concentration of salidroside in rat plasma was over the range of calibration curve, appropriate dilutions were applied to the plasma sample with blank rat plasma before sample processing. 

### 3.5. Method Validation

The method for simultaneous determination of salidroside and *p*-tyrosol in rat plasma by LC-MS/MS was validated to meet the acceptance criteria as the guidance from Food Drug Administration (FDA) and per guidelines of the International Conference on Harmonization of Technical Requirements for Registration of Pharmaceuticals for Human Use (ICH). 

The selectivity was assessed by comparing the chromatograms of six individual blank rat plasma samples with the corresponding spiked plasma samples. Each blank plasma sample was processed through the proposed extraction procedure and tested to ensure no interference of the analyte from the rat plasma.

Lower limit of quantification (LLOQ) was defined as 10 times the signal-to-noise (S/N) ratio, and limit of detection (LOD) was done as three times the S/N ratio. The lowest concentration on the calibration curve was to be accepted as the LLOQ, if the analyte response was at least 10 times more than that of blank plasma sample. 

Linearity was assessed by assaying calibration curves ranging from 50 to 2,000 ng/mL for salidroside and 20–200 ng/mL for *p-*tyrosol in duplicate on six consecutive days. Plasma samples were quantified using the peak area ratio of salidroside or *p-*tyrosol to that of IS standard curves are in the form of *y* = A + B*x*, where y represents the plasma concentration of analyte and *x* represents the ratio of analyte peak area to that of IS The acceptance criterion for a calibration curve was a correlation coefficient (r) of 0.99 or better, and each back-calculated standard concentration must be within 100 ± 15% except at LLOQ, for which the maximum acceptable deviation was at 20%. 

Accuracy and precision were evaluated at three QC levels of 50, 500 and 2,000 ng/mL for salidroside and of 20, 100, 200 ng/mL for *p-*tyrosol. The assays of intra- or inter-day accuracy were preformed in six separate runs on the same day or on six consecutive days, and expressed as (observed concentration/spiked concentration) ×100%. Intra- and inter-day precisions were obtained by one-way analysis of variance (ANOVA) test, and were expressed as relative standard deviation (RSD). The accuracy was required to be within 100 ± 15% and the precision should not exceed ±15%.

The extraction recoveries of salidroside and *p-*tyrosol were determined at three QC levels, respectively. Recoveries were calculated by comparing the analyte/IS peak area ratios of each analyte in spiked plasma samples with those of analytes in the matrices by spiking extracted analyte-free plasma samples prior to chromatography.

The matrix effects from endogenous substances present in extracted rat plasma may cause ion suppression or enhancement of the signal. The matrix effects were investigated by post-extraction spike method in the present study. Peak area (A) of standard analyte in spiked blank plasma was compared with the corresponding peak area (B) obtained by directly injecting the standard analyte in the mobile phase at concentrations of 50, 500 and 2,000 ng/mL for salidroside and 20, 100 and 200 ng/mL for *p-*tyrosol in triplicates. The peak area ratio of A/B (as a percentage) was used as a quantitative measure of the matrix effects [33].

### 3.6. Stability

The stability of standard solutions was tested at room temperature for 2 h and upon refrigeration (4 °C) for 30 days. The stability of analytes was examined by keeping replicates of salidroside and *p*-tyrosol QC samples in the autosampler tray for 24 h and in a freezer at −20 °C for 30 days; the freeze-thaw stability was obtained over three freeze-thaw cycles, by thawing at room temperature for 2–3 h and then refreezing at −20 °C for 12–24 h. For each concentration and each storage condition, 6 replicates were analyzed in one analytical batch. The concentration of analytes after each storage period was related to the initial concentration, which was determined when the samples were originally prepared and processed. 

### 3.7. Pharmacokinetic and Bioavailability Studies

Male rats (ICR, 200 ± 20 g) were obtained from Laboratory Animal Center of Jilin University (Changchun, Jilin Province, China). Animal handling procedures were according to standard operating procedure approved by the institutional animal care and use committee. All rats were dosed following an overnight fasting (except for water).

For the identification of salidroside and *p*-tyrosol, six male rats were divided into two groups. Rats in one group underwent an i.v. administration through the vena caudalis with sodium chloride solution of salidroside at the dose of 50 mg of salidroside (12.5 mg/mL in saline) per kg body weight (i.v. 50 mg/kg). Five min after administration, blood samples were collected in heparinized tubes via cardiac puncture. Rats in the other group received an i.g. dose of 100 mg of salidroside (25 mg/mL in saline) per kg body weight (i.g. 100 mg/kg), and blood samples were collected at 0.5 h after administration. Plasma was separated by centrifuging at 4,000 g for 30 min at 4 °C and stored frozen at −20 °C until analysis.

For pharmacokinetic study, 12 male rats underwent jugular vein cannulation [34] and were randomly divided into two groups. Rats in group 1 were i.v. administered with 50 mg/kg. Serial blood samples (about 0.3 mL) were collected in heparinized tubes via the jugular vein before and at the time points of 0, 0.08, 0.17, 0.33, 0.5, 1.0, 1.5, 2.0, 3.0, 4.0 h after administration. In group 2, each rat received the administration of i.g. 100 mg/kg. Blood samples were collected before and at the time points of 0, 0.08, 0.17, 0.25, 0.33, 0.5, 0.75, 1.0, 1.5, 2.0, 3.0, 4.0, 6.0, 8.0 h. Plasma was separated and stored frozen at −20 °C until analysis.

A noncompartmental pharmacokinetic analysis using Kinetica^TM^ software package (version 5.0, Thermo Fisher Scientific Inc., Pittsburgh, PA, USA) was performed to determine the key parameters including maximum concentration (C_max_), time-to-maximum concentration (T_max_), half life time (T_1/2_), total body clearance (Cl), mean residence time (MRT), steady state apparent volume of distribution (Vss), area under curve from zero to the last measurable plasma concentration point (AUC_0−t_, t = 4.0 h for i.v. administration, t = 8.0 h for i.g. administration), and area under the plasma concentration-time curve from zero to time infinity (AUC_0–∞_). 

The oral bioavailability (F) is defined as the fraction of unchanged drug reaching the systemic circulation following administration through the i.g. route. The absolute oral bioavailability of a drug is generally measured by comparing the respective AUCs after i.g. and i.v. administration according to the following equation [28]: 


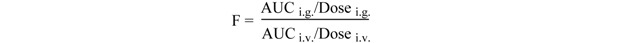


## 4. Conclusions

The aglycone metabolite of salidroside, *p*-tyrosol, was identified after i.v. administration at a dose of 50 mg/kg, but was not detectable after i.g. administration which was confirmed by the HPLC-PDA and LC-MS/MS analysis. Then, an accurate and precise LC-MS/MS method was developed and validated to quantitatively determine salidroside and *p*-tyrosol in rat plasma samples. The method has been successfully applied to pharmacokinetic studies, and the oral bioavailability was calculated. 
